# Incorporation of interfacial roughness into recursion matrix formalism of dynamical X-ray diffraction in multilayers and superlattices^1^

**DOI:** 10.1107/S1600576717004137

**Published:** 2017-04-13

**Authors:** Ihar Lobach, Andrei Benediktovitch, Alexander Ulyanenkov

**Affiliations:** a Atomicus OOO, Minsk, Belarus; b Atomicus GmbH, Karsruhe, Germany

**Keywords:** interfacial roughness, transition layers, multilayers, dynamical diffraction, recursion matrix formalism

## Abstract

Diffraction in multilayers in the presence of interfacial roughness is studied theoretically, the roughness being considered as a transition layer. Exact (within the framework of the two-beam dynamical diffraction theory) differential equations for field amplitudes in a crystalline structure with varying properties along its surface normal are obtained. An iterative scheme for approximate solution of the equations is developed. The presented approach to interfacial roughness is incorporated into the recursion matrix formalism in a way that obviates possible numerical problems. Fitting of the experimental rocking curve is performed in order to test the possibility of reconstructing the roughness value from a diffraction scan. The developed algorithm works substantially faster than the traditional approach to dealing with a transition layer (dividing it into a finite number of thin lamellae). Calculations by the proposed approach are only two to three times longer than calculations for corresponding structures with ideally sharp interfaces.

## Abstract 2

Interfacial roughness is considered as a transition layer. A method of calculation of diffraction scans from multilayered structures with interfacial roughness, which is both fast and free of numerical errors, is developed.

## 1. Introduction   

The phenomenon of interfacial roughness in multilayers has been shown to have an influence on the functioning of electronic devices (*e.g.* see Zolotoyabko, 1998 ▸; Bolognesi *et al.*, 1992 ▸; Ming *et al.*, 1993 ▸; People & People, 1986 ▸; People *et al.*, 1984 ▸; Glaser *et al.*, 1990 ▸). Therefore it is important to have a means to estimate this nonideality of interfaces. In the case of grazing incidence in X-ray reflection (XRR), when most of the reflected power is produced by surface layers, interfacial roughness considerably affects measured XRR scans and has been extensively studied by this technique (*e.g.* see Feranchuk, Feranchuk *et al.*, 2003 ▸; de Boer, 1994 ▸, 1995 ▸, 1996 ▸; Feranchuk *et al.*, 2007 ▸; Sinha *et al.*, 1988 ▸; Feranchuk, Minkevich & Ulyanenkov, 2003 ▸; Benediktovitch *et al.*, 2014 ▸; Pietsch *et al.*, 2013 ▸).

In X-ray diffraction (XRD), just like in XRR, grazing-incidence geometry is sensitive to interfacial roughness (see Stepanov & Köhler, 1994 ▸). One may expect that in conventional geometries, for example 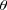
–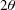 scans, the roughness effect will be subtle. This is true in many cases; however, sometimes scans are sensitive to interfacial roughness even in conventional geometries, for example for superlattices consisting of thin layers (Fullerton *et al.*, 1992 ▸). This is why we consider interfacial roughness in conventional XRD geometry in this paper.

It is possible to treat interfacial roughness as a transition layer (see Stepanov & Köhler, 1994 ▸) by dividing it into many thin lamellae. However, this approach turns out to be very time consuming. Therefore, we decided to speed up the process by performing analytical calculations for transition layers and incorporation of interfacial roughness into the recursion matrix formalism (RMF) (see Stepanov *et al.*, 1998 ▸). We will show that it is possible to introduce only one additional matrix for each layer, describing the roughness of this layer. This boosts the speed of calculation, which is important for fitting of experimental curves (Ulyanenkov & Sobolewski, 2005 ▸).

This paper is organized in the following way. In §2, basic notations for the two-wave dynamical diffraction theory (DDT) in multilayered structures are introduced, which allow the transition layer to be treated as many thin lamellae (traditional numerical approach; see Stepanov & Köhler, 1994 ▸). In §3, several types of transition layers are analyzed and one of them is selected for further consideration. In §4, we obtain differential equations for field amplitudes in continuous transition layers by assuming that the thickness of each lamella tends to zero. A natural ansatz for solution of the equations is proposed and a useful approximate solution is found. In the framework of this approach, it is shown that the effect of interfacial roughness can be described by an additional matrix in the RMF approach. §5 provides numerical examples and illustrates the fitting of one experimental rocking curve.

## 2. Summary of two-beam DDT in multilayered samples   

**Figure 1:**
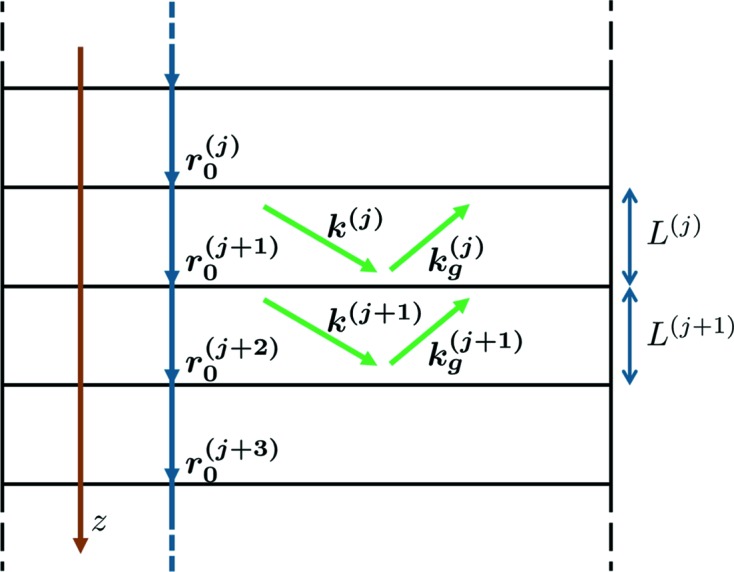
Sketch of the multilayered sample. 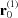 lies on the surface of the sample.

The structure considered in this section is presented in Fig. 1 ▸. In order to describe a transition layer by this structure one has to choose a change of Fourier components of susceptibility 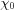, 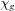, 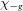 from layer to layer, such that the phase of oscillation of electron density does not encounter any jumps in the interface between neighboring layers. One can check by direct substitution that the following expression for the susceptibility in the *j*th layer is in agreement with the last requirement (Stepanov *et al.*, 1998 ▸): 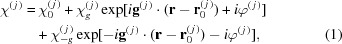
with 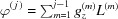 (layers are numbered by 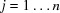). 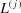 is the thickness of the *j*th layer and 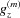 represents the *z* component of the reciprocal lattice vector in the *m*th layer 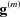. For 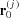 see Fig. 1 ▸. In equation (2) 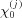, 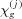 and 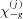 are supposed to have the same phase as 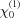, 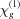 and 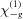, respectively. Actually, these phases change slightly if one describes the transition between two real crystals owing to nonzero absorption, but this error is insignificant.

The problem of dynamical diffraction in multilayered structures has been solved many times and we only present final results for the electric field in the layers for the case when specularly reflected waves can be neglected. We consider σ polarization only, because the results for π polarization can be readily obtained by multiplying 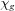 and 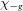 by the polarization factor 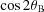, where 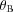 is the Bragg angle. The solution for the Fourier transform (over time) of the electric field in the *j*th layer is (see *e.g.* Stepanov & Köhler, 1994 ▸)
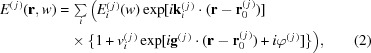
where *w* is the angular frequency in the Fourier transform and 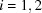. 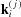 represents the wavevector of the direct wave corresponding to the solution of the dispersion equation in the *j*th layer (in general, there are four solutions to the dispersion equation, but two of them correspond to negligible specularly reflected waves). 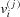 is the ratio of amplitudes of diffracted and direct waves in the *j*th layer. It is given by 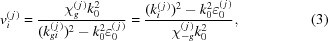
where 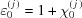. 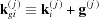 represents the wavevector of the diffracted wave and 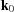 is the wavevector of the incident wave. Actually, the component of the wavevector of the direct wave parallel to the crystal surface is fixed during solution of the dispersion equation. Therefore, we will call the normal component of the vector 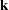 a solution of the dispersion equation. Hereinafter we denote the attenuating root by 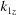 [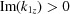] and the amplifying one by 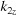 [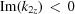].

The boundary conditions in the interface between the *j*th and (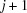)th layers can be written as continuity of the electric field (see Benediktovitch *et al.*, 2014 ▸) separately for the waves with components parallel to the surface equal to 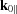 and (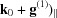: 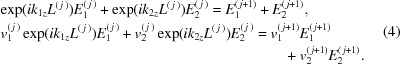
Now let us switch to matrix notation, similar to that of Stepanov *et al.* (1998 ▸). We introduce a column vector of field amplitudes and two auxiliary matrices: 
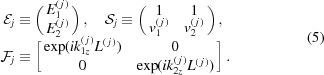
Making use of these matrices, equations (4) take the form 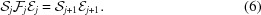
Even though now the equations look rather explicit, let us try to simplify them further by introducing new unknown amplitudes as a linear combination of current ones: 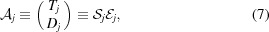
where *T* stands for ‘transmitted’ and *D* stands for ‘diffracted’. 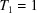 is the amplitude of the incident wave and 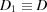 is the amplitude of the wave diffracted from the whole sample. If we denote the vacuum below the sample as the 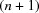
th layer, then 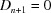, which means that no wave is incident on the lower surface of the sample in the direction opposite to the *z* axis. For the introduced amplitudes the following relations are true:
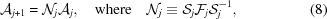


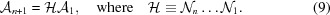
If we rewrite equation (9) in more detail (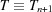), 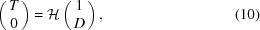
it becomes apparent that it is a linear system for unknown amplitudes of diffracted (*D*) and transmitted (*T*) waves. *D* can be expressed as
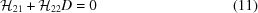
or, if we divide it by the increasing exponents 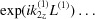 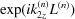, 
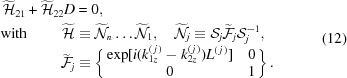
In this way, the final expression for *D*, 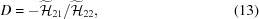
does not contain any increasing exponents. Thus, consideration of thick layers will cause no numerical problems. Note that the approach presented here of precluding numerical errors is different from that reported by Stepanov *et al.* (1998 ▸). Clearly, one can consider any set of layers using the presented approach, not only transition layers. If one considers the more realistic problem where the 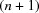
th vacuum layer is missing and the *n*th layer is the substrate (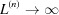), then 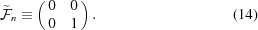



## 3. Choice of transition layer model   

In this section we consider several types of transition profiles and their influence on the diffracted intensity distribution. We investigate the following three types of transition profiles:

Type a
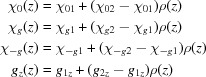



Type b
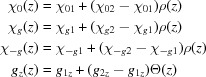



**Figure 2:**
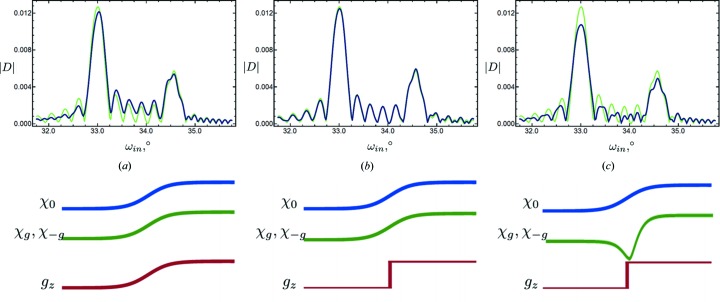
Symmetric scans of the magnitudes of the amplitude of the diffracted wave from a sample (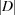) consisting of two layers (Ge and Si) of the same thickness (20 nm) for types of transition layers (*a*) a, (*b*) b and (*c*) c. Reflection 004, roughness parameter σ = 2 nm. 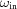 is the angle of incidence (with respect to the sample’s surface). Light-green and dark-blue lines correspond to zero and nonzero roughness, respectively. Under the plots a qualitative description of the susceptibilities near the interface as a function of *z* is given.

Type c
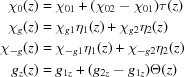
with
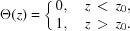
Additional indices 1 and 2 describe the properties of actual upper and lower layers, and 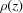, 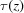 and 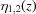 are smooth functions providing the behavior shown in the lower part of Fig. 2 ▸:
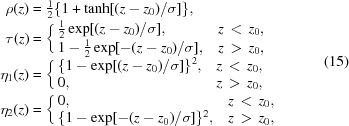
where σ is the quantitative characteristic of roughness and 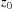 is the *z* coordinate of the interface between two actual layers (*z* of the upper surface of the sample is zero).

Type a (the Epstein profile; Epstein, 1930 ▸) causes an increase in diffracted intensity between the peaks (see Fig. 2 ▸ *a*). The reason is that, if 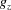 changes smoothly, then at every angle of incidence (within the region between two peaks of different actual layers) there are sublayers in the transition layer for which the Bragg condition is fulfilled and they provide higher diffracted amplitude. In our opinion, the effect of interfacial roughness is likely to reduce the occurrence of diffraction in the lamellae close to the interface, rather than to shift it to other values of the incidence angle.

In cases of type b, the *z* component of 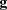 changes stepwise and, as is seen in Fig. 2 ▸(*b*), the increase between the peaks disappears. However, near the left peak, one can see an enhancement, which is connected to the specific choice of dependence of 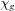 and 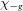 on *z*.

Type c (see Fig. 2 ▸ *c*) can be understood as follows: The abrupt change of the 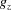 profile corresponds to an interface at which the average coherent lattice periodicity is changed. The drop in the susceptibilities 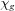 and 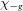 corresponds to a reduction of crystallographic order due to the static Debye–Waller factor conditioned by defects located at the interface. Such a situation can occur, for example, for the system described by Satapathy *et al.* (2005 ▸) where the nonuniform strain decays exponentially when moving away from the boundary. This type will be used further.

## 4. Differential equations for field amplitudes in transition layers: approximate solution   

In the previous section we dealt with the problem of the transition layer by dividing it into many finite sublayers, and the solution for a field inside the sample was given by 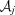, 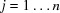. Now we consider division into infinitesimal sublayers. In this case the solution will be a function 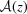 [we will omit ‘(*z*)’ from now on], and the boundary conditions at all interfaces will be replaced by differential equations. Let us obtain them. Consider equation (8) in the case of infinitesimal sublayers. Making the substitutions 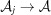, 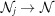, 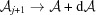 in equation (8) results in 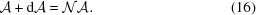
Further, it is necessary to take into account that under the assumption of infinitesimal sublayer thickness 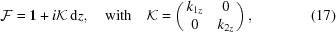
where indices *j* are omitted and the dependence on *z* is assumed. Also, 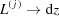. By equation (8) with 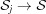 we obtain 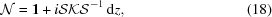
and by equation (16)

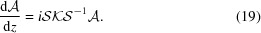
Equation (19) is the differential equation for field amplitudes in a crystalline sample whose properties vary along the *z* direction. Its domain of applicability coincides with that of two-beam DDT with two diffraction roots [diffraction in each infinitesimal sublayer of the sample should be properly described by DDT in order that equation (19) be correct].

Our present goal is to find how roughness influences the transition matrix of one layer in a structure. In the case of nonzero roughness it is unclear what an interface is. We introduce it formally as the point where 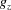 jumps from one value to the other (as in type c). As we consider one layer, we choose new coordinates, where 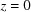 in the upper interface. The thickness of the layer is *L*, the susceptibilities not perturbed by roughness are 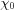, 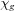 and 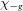, and the susceptibilities perturbed by roughness are 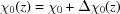, 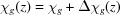 and 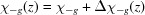. We will also introduce a function 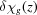: 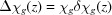, 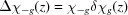. The reciprocal lattice vector is 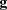 and is constant. Note that by the last definitions we can consider roughness in both the upper and the lower surfaces of the layer (corresponding modifications to type c will be made below). Further, we make ordinary approximations of two-beam DDT (search for roots in the vicinity of 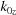) and obtain an expression for the matrix 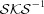. Then, after some algebra, equation (19) takes the form 
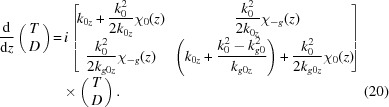


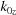
 is the *z* component of 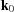, which is the wavevector of the incident wave. Similarly, 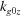 is the *z* component of 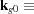 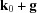. Equations (20) are similar to Takagi–Taupin equations (Takagi, 1962 ▸, 1969 ▸; Taupin, 1964 ▸). However, the validity of the obtained equations is not restricted to a smooth variation of susceptibilities. In the case of constant susceptibilities, equation (20) can be solved by Euler’s method, searching for a solution in the form of exponents, and the solution is as follows: 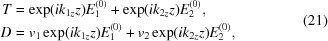
which is precisely what one would expect in the case of an ideal crystal. 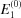 and 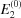 are constants of integration. The meanings of 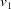, 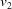, 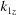 and 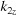 are the same as in equations (3) and (4) and they are expressed through 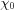, 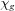, 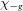, 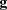 and 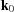.

We will seek a solution of equation (20) in the form of equations (21) with varying 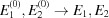. Substitution of the ansatz into equation (20) gives the following:
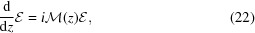
with 
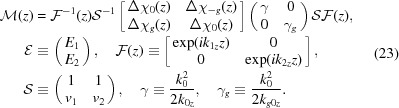
One can see that if 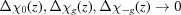 then the solution is 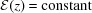. Equation (22) has an exact analytical solution in the case that 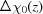, 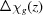 and 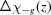 are linear functions of 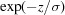, where σ is the roughness parameter. There is a plausible model consistent with the last requirement, but the solution is cumbersome and has numerical problems. It is expressed through the Laguerre polynomial and hypergeometric function (not shown here). However, it appears that an approximate solution to equation (22) is very accurate at realistic values of roughness. The relative error with respect to the exact solution is comparable to the accuracy of two-beam DDT. Namely, one can find a first-order approximation 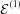 by integration of equation (22), assuming 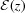 is constant on the right-hand side: 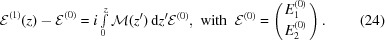
In further consideration we will need the value of 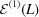:
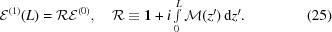
We call 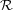 the roughness matrix. Let us now consider equation (6) as the boundary conditions at the interface between two actual layers. One has the field at the lower interface of the *j*th layer on the left-hand side. In the case of nonzero roughness this field can be expressed through the above-mentioned ansatz using equation (25). Then equation (6) takes the form 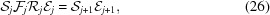
where 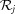 is the roughness matrix of the *j*th layer. Further, it is convenient to denote 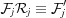 and obtain the diffracted amplitude from a multilayer structure of actual layers by equation (13), having made the substitution 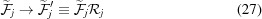
in equations (12). Thus, the formal solution to the problem with nonzero roughness is found.

**Figure 3:**
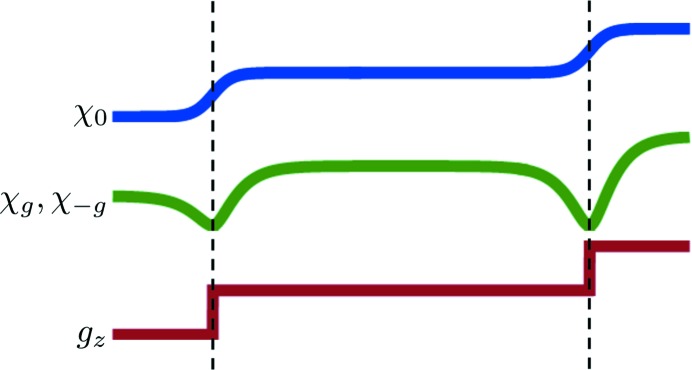
Qualitative description of type c*. The layer under consideration is placed between the two dashed lines.

Below we will find an expression for 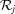 and 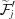 in the case of a transition layer of type c. Let us modify it to account for roughness in both interfaces of the layer:

Type c*
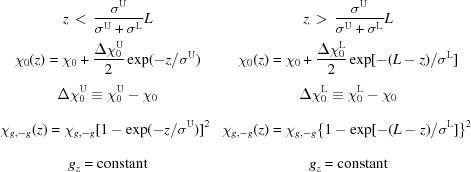
where upper indices U and L imply the layer above or below the one under consideration (see Fig. 3 ▸).

In this case 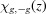 are continuous functions and 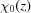 is almost continuous. Actually, a small discontinuity has a subtle influence as only the integrals of these functions will be used. Performing the integration in equation (25) in the case of type c* one obtains 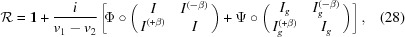
where ‘
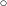
’ means the Hadamard product, that is 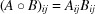 (no summation over *i* and *j*). 
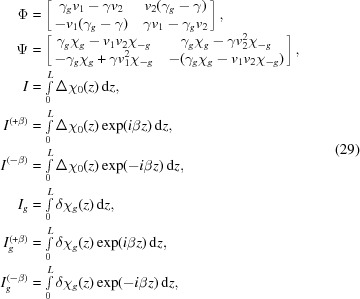
with 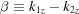. However, in the final equation for the amplitude diffracted from the sample one needs an expression for 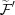: 
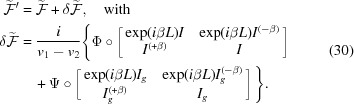
This is expressed *via* the following quantities (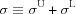): 
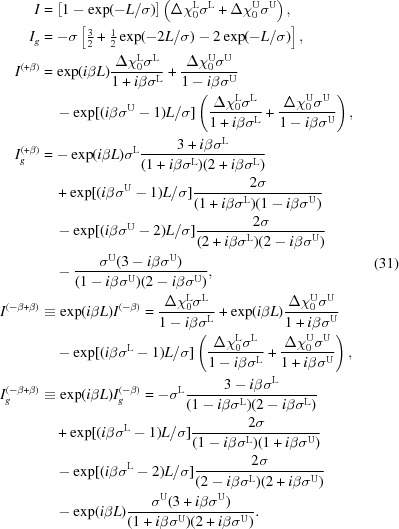
It appears that 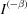 and 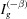 diverge with 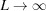. However, the final expression for 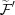 is not divergent since it involves the convergent quantities 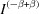 and 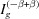 in equations (31). Thus, to avoid numerical problems one should use 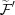 of equation (30) using expressions from equations (31). We can check the limiting case 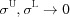. It is seen from equations (31) that this leads to 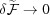 as well. 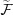 for the substrate layer is readily obtained by considering the limit 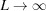 in equations (31) and (30). Lastly, some expressions in equations (31) are convergent only if 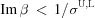. However, this condition is not fulfilled only at very large values of the roughness parameter (hundreds of nanometres). The derivation of the roughness matrix for amorphous layers is analogous and also much simpler than for crystalline layers. Therefore, we do not present it here.

## 5. Numerical examples and fitting of experimental rocking curve   

**Figure 4:**
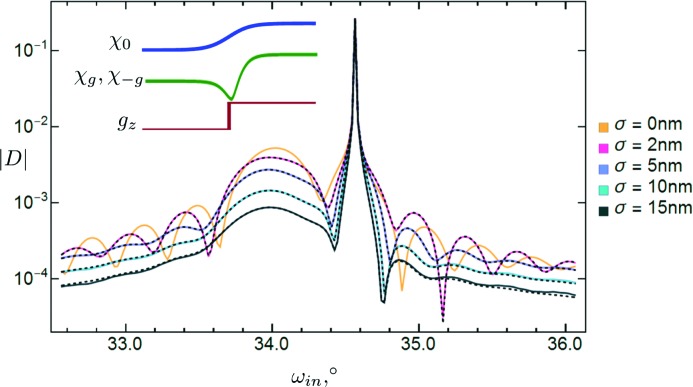
Calculated symmetric scans of a thin film (15 nm) of Ge solution (10%) in Si on an Si substrate. Reflection 004. Type c of transition layer model is assumed. 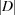 is the magnitude of the amplitude of the diffracted wave (σ polarization) and 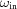 is the incidence angle (with respect to the surface plane). The light-orange solid line represents the case of zero roughness. Pink, purple, cyan and black solid lines correspond to interfacial roughnesses of 2, 5, 10 and 15 nm, respectively (surface roughness is absent). These colored lines depict the exact solution. Related black dashed lines represent the approximate solution.

Let us prove that the suggested approximate solution is accurate. A comparison of exact and approximate scans for a simple test sample is given in Fig. 4 ▸. Hereafter, by exact solution we mean the one obtained by division of the sample into many thin lamellae of finite thickness. The values of the roughness parameter are very large in this example (up to the thickness of the upper layer). They are chosen in this way in order to find the limits of applicability of the approximate solution. In Fig. 4 ▸ one can see that the dashed lines start to deviate slightly from the solid lines at values of the roughness parameter of about 10 nm. For realistic values (up to several ångströms) the exact and approximate lines are indistinguishable, which proves the reliability of the approximate solution.

**Figure 5:**
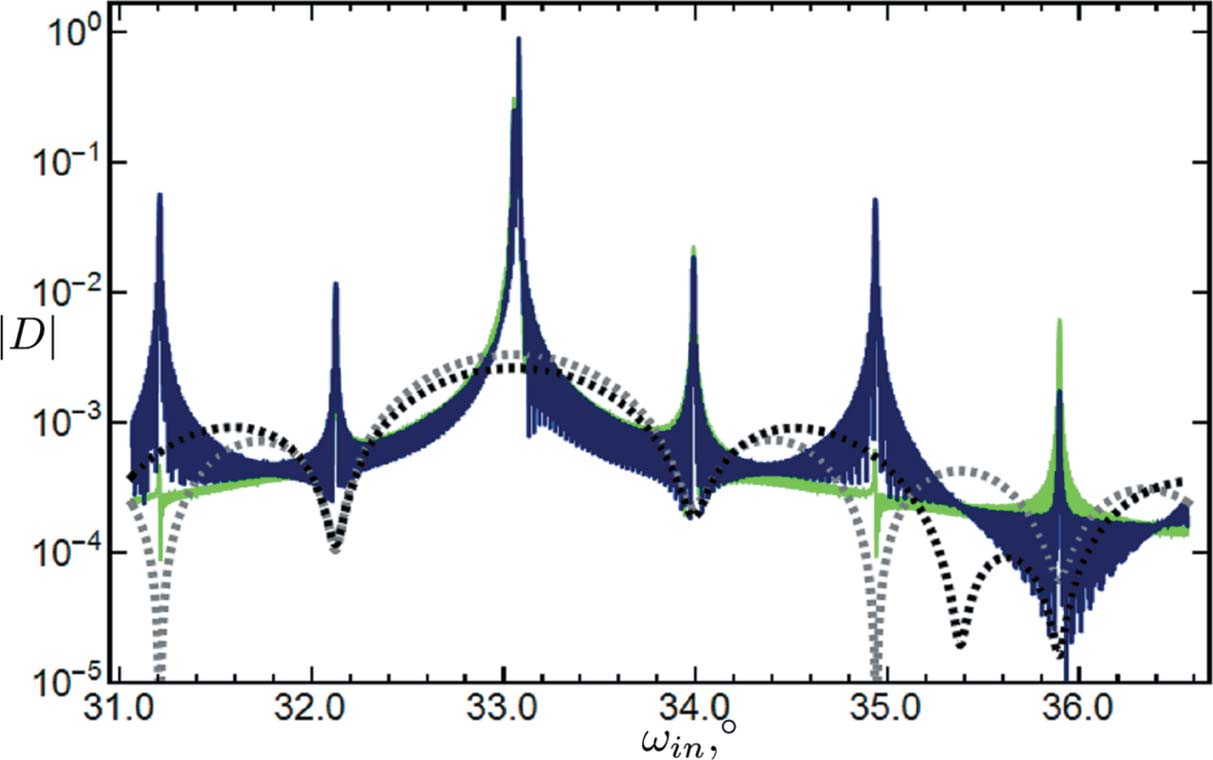
Calculated symmetric scans of a superlattice [GaAs(2.82271 nm)/Al
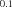
Ga
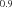
As(2.82675 nm)]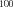 on a GaAs substrate. Reflection 004, interfacial roughness 0.2 nm, σ polarization. 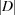 is the magnitude of the amplitude of the diffracted wave and 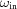 is the incidence angle (with respect to the surface plane). Type c of transition layer model is assumed. Each interface has roughness. The dark-blue scan takes into account roughness, and the light-green one does not. Black and gray dashed lines represent scans of one period of the superlattice with and without interfacial roughness, respectively.

Fig. 5 ▸ shows calculated scans of a superlattice based on the type c transition layer model. Note that some peaks are enlarged in the case of nonzero roughness, whereas others are reduced. Actually, this is in accordance with diffraction in each period of the superlattice. That is, if the diffracted amplitude from one period is decreased owing to roughness at a certain incidence angle, then the amplitude of the wave diffracted from the superlattice is reduced too and *vice versa*.

**Figure 6:**
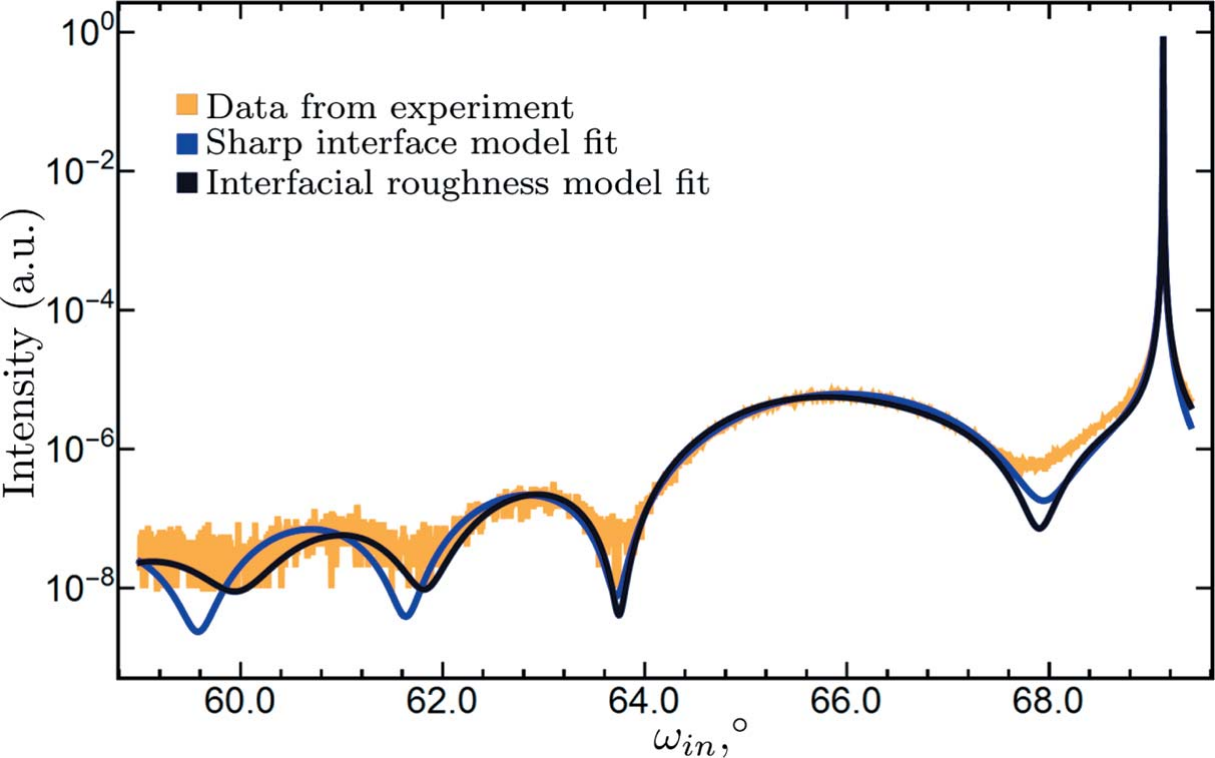
A thin layer (5 nm) of Si
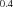
Ge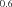 on an Si substrate. Reflection 004. Symmetric scan. The blue line represents the calculation for the nominal model of the structure with sharp interfaces. The black line depicts the fit by the model with interfacial roughness. In the fit, the thickness is 5.8 nm and the roughness is 0.6 nm.

We also performed a test fitting of an experimental curve by the proposed method of calculation of rocking curves taking into account roughness (see Fig. 6 ▸). In addition, the fitting was done by the conventional nominal model with ideally sharp interfaces. In the case of the model with roughness, the variable parameters were the vertical scale, the layer’s thickness, and the roughness parameter at the interface between the layer and the substrate. In the case of the nominal model, the only variable parameter was the vertical scale; the layer’s thickness was fixed and equal to 5 nm (this follows from the period of thickness fringes). The best fit was determined by finding the curve with the smallest squared deviation (in logarithmic scale) from the experimental rocking curve. The experimental curve was taken from the work of Ulyanenkova *et al.* (2013 ▸).

The area near the substrate peak where the largest discrepancy is observed is dominated by the shape of the substrate peak. Since it is much higher in intensity than the layer, the deviation of its shape from DDT predictions affects the curve significantly. The deviations can be due to instrumental effects or due to the influence of defects [see, for example, the paper by Shreeman & Matyi (2010 ▸), where the fit of the region near the substrate for a similar structure was obtained in the frame of statistical dynamical diffraction theory].

In our case we are within the DDT formalism and the focus was on the intensity from the layer. Hence, the left part of the profile where the thickness fringes are visible is of interest. Here we can see that the interfacial roughness model follows the curve closer than the sharp interface model.

## 6. Conclusions   

In this paper we obtained a general result, the application of which is not limited to interfacial roughness. We presented the exact (in the framework of DDT) equations for wave amplitudes in a crystal whose properties vary along its surface normal [see equation (20)]. These equations could be potentially applied to many other problems apart from interfacial roughness. For instance, one could use these equations in crystals with strain along the surface normal. This might be a subject of further research.

Another independent result is that equation (20) has an exact solution for a plausible model of the transition layer describing interfacial roughness [when the Fourier components of susceptibility are linear functions of 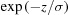]. However, this solution is impractical because it engenders copious numerical problems. This is why it was not exploited in this paper.

Nevertheless, we have developed an approximate iterative method of solution of equation (20) [see equations (24) and (25)]. In the numerical examples in §5, it is shown that for realistic values of the roughness parameter the approximate solution is indistinguishable from the exact one. However, in practice it is calculated much faster than the exact one. The speed is comparable (two to three times longer) to that of corresponding calculations in the model with ideally sharp interfaces. As to the lamellar approach, to achieve sufficient accuracy we had to use about 100 lamellae and this made the calculations several hundred times longer with regard to the ideal interface model. Generally, the roughness matrix approach improves the speed of calculations by about a factor of *N*, where *N* is the number of lamellae used to model the transition layer. Clearly, this is a boon when performing fitting of experimental rocking curves. The method of taking into account roughness was incorporated into RMF in multilayers [see equation (26)]. In order to take advantage of the speed of the approximate solution one has to calculate analytically an explicit form of the roughness matrix for the used model of the transition layer. The results of such a calculation for the model considered in this paper are given in equations (28), (30) and (31).

A trial fitting of the experimental curve showed that the roughness matrix approach with transition layer model type c provides better agreement with experimental rocking curves in the region of thickness fringes far from the peak of the substrate. In the vicinity of the substrate both models (nominal and type c) deviate from the experimental curve. The disagreement may be caused by instrumental effects or by the influence of defects. However, this single fit does not provide enough evidence to make any final conclusions. In the future, additional experiments should be performed, possibly with superlattices, where interfacial roughness is thought to have a stronger influence on rocking curves.

It would also be desirable to make some additional independent measurements of the roughness of studied samples, for example by XRR or electron microscopy.
